# *Bean pod mottle virus*: a new powerful tool for functional genomics studies in *Pisum sativum*

**DOI:** 10.1111/pbi.12537

**Published:** 2016-02-20

**Authors:** Chouaib Meziadi, Sophie Blanchet, Manon M.S. Richard, Marie‐Laure Pilet‐Nayel, Valérie Geffroy, Stéphanie Pflieger

**Affiliations:** ^1^ Institute of Plant Sciences Paris‐Saclay (IPS2)CNRSINRAUniversité Paris‐SudUniversité d'EvryUniversité Paris‐Diderot Sorbonne Paris‐CitéUniversité Paris‐SaclayOrsayFrance; ^2^ INRAUMR 1349 IGEPPLe RheuFrance

**Keywords:** *Pisum sativum*, functional validation, RNAi, post‐transcriptional gene silencing, legume, *Bean pod mottle virus*

## Abstract

Pea (*Pisum sativum L*.) is an important legume worldwide. The importance of pea in arable rotations and nutritional value for both human and animal consumption have fostered sustained production and different studies to improve agronomic traits of interest. Moreover, complete sequencing of the pea genome is currently underway and will lead to the identification of a large number of genes potentially associated with important agronomic traits. Because stable genetic transformation is laborious for pea, virus‐induced gene silencing (VIGS) appears as a powerful alternative technology for determining the function of unknown genes. In this work, we present a rapid and efficient viral inoculation method using DNA infectious plasmids of *Bean pod mottle virus* (BPMV)‐derived VIGS vector. Six pea genotypes with important genes controlling biotic and/or abiotic stresses were found susceptible to BPMV carrying a GFP reporter gene and showed fluorescence in both shoots and roots. In a second step, we investigated 37 additional pea genotypes and found that 30 were susceptible to BPMV and only 7 were resistant. The capacity of BPMV to induce silencing of endogenes was investigated in the most susceptible genotype using two visual reporter genes: *PsPDS* and *PsKORRIGAN1* (*PsKOR1*) encoding *PHYTOENE DESATURASE* and a 1,4‐β‐D‐glucanase, respectively. The features of the ‘one‐step’ BPMV‐derived VIGS vector include (i) the ease of rub‐inoculation, without any need for biolistic or agro‐inoculation procedures, (ii) simple cost‐effective procedure and (iii) noninterference of viral symptoms with silencing. These features make BPMV the most adapted VIGS vector in pea to make low‐ to high‐throughput VIGS studies.

## Introduction

Pea (*Pisum sativum* L.) belongs to the *Fabaceae* plant family. Pea is a cool‐season legume crop, which produces seeds of high nutritional value for feed and food that are an excellent protein complement of cereal‐based diets. In Europe and especially in France, pea is currently the major grain legume crop and is mainly cultivated for the production of forage for animal consumption. But since the end of the nineties, the development of this crop has gradually decreased due to yield inconsistency. Consequently, European countries import about 70% of their plant protein consumption. Thus, significant productivity progress has to be made and stable and high‐yielding varieties are needed.

Different biotic and abiotic stresses are responsible for yield instability. First, root rot disease caused by the oomycete *Aphanomyces euteiches*, considerably reduces the yields of spring pea (Kraft and Pfleger, 2001). Genetic resistance to *A. euteiches* root rot was shown to be partial and controlled by multiple genes (Hamon *et al*., 2010, 2013; Pilet‐Nayel *et al*., 2002, 2005). In winter peas, early sowings can increase yields and limit the effect of late‐stage stresses, but cultivars need to have a high level of frost tolerance, a complex genetic trait (Lejeune‐Henaut *et al*., 2008). Moreover, Ascochyta blight caused by a complex of three fungal pathogens affects aerial plant parts and can have devastating effects on yield of winter peas (Kraft and Pfleger, 2001). Both major genes and quantitative trait loci, conferring resistance to Ascochyta blight, have been identified (Clulow *et al*., 1991; Darby *et al*., 1985; Fondevilla *et al*., 2011; Prioul *et al*., 2004; Tar'an *et al*., 2003; Timmerman‐Vaughan *et al*., 2002, 2004). Many genes controlling frost tolerance and resistance to *A. euteiches* root rot and Ascochyta blight have been mapped on the consensus genetic map of pea (Hamon *et al*., 2010, 2013; Lejeune‐Henaut *et al*., 2008; Pilet‐Nayel *et al*., 2002, 2005; Prioul *et al*., 2004). To identify the underlying gene sequences, fine mapping of the corresponding regions is needed for the identification of candidate genes that must be further validated using functional genetics tools. Ultimately, the discovery of gene functions will facilitate the genetic improvement of commercial pea varieties by molecular breeding.

Furthermore, with the future availability of the whole‐genome sequence of pea (Alves‐Carvalho *et al*., 2015; McGee, 2013), it is essential for the pea research community to develop novel molecular tools for large‐scale analysis of gene functions at the genome‐wide level. Although pea is amenable to stable genetic transformation (Somers *et al*., 2003; Svabova *et al*., 2005), this remains a challenge and thus hampers the application of conventional reverse genetics approaches such as T‐DNA insertion mutagenesis (AzpirozLeehan and Feldmann, 1997) and RNA interference (McGinnis *et al*., 2005). In pea, fast neutron and TILLING mutant populations have been developed (Dalmais *et al*., 2008; Hofer *et al*., 2009) but to perform large‐scale, high‐throughput genomic studies in pea, a cheap and rapid system that bypasses genetic transformation is required.

Virus‐induced gene silencing (VIGS) is an attractive reverse genetics tool for functional genomics in plants. VIGS exploits a plant innate antiviral defence that is based on post‐transcriptional gene silencing (PTGS) (Kumagai *et al*., 1995). In this defence mechanism, the presence of viral‐derived double‐stranded RNA (dsRNA) molecules triggers Dicer‐like (DCL) proteins that identify and cleave dsRNA, generating 21–23 nucleotide viral‐derived short‐interfering RNAs (siRNAs). These siRNAs in association with the RNA‐induced silencing complex (RISC), target homologous viral RNA for degradation. In VIGS, a recombinant virus carrying a fragment of a specific host gene is first constructed and inoculated in susceptible host plants. PTGS is triggered causing the sequence‐specific degradation of mRNA of the target gene resulting in a knockdown phenotype that can be used to assess gene function (Burch‐Smith *et al*., 2004; Robertson, 2004).

To date, several VIGS vectors have been designed to address reverse genetics needs in various plants (Lange *et al*., 2013). In legumes, VIGS vectors were developed from several plant viruses, such as the *Apple latent spherical virus* (ALSV, genus *Cheravirus*), the *Pea early browning virus* (PEBV, genus *Tobravirus*) and the *Bean pod mottle virus* (BPMV, genus *Comovirus*, family *Comoviridae*) (reviewed in Pflieger *et al*., 2013). In pea, PEBV was originally developed for VIGS studies (Constantin *et al*., 2004), but its use is restricted to low‐throughput analyses because delivery of the viral vector into plants is not possible by mechanical inoculation and requires agro‐inoculation which may become laborious and time‐consuming for high‐throughput analyses (Pflieger *et al*., 2013).

In legumes, the most adapted VIGS vector for high‐throughput genomics studies is the ‘one‐step’ VIGS vector derived from BPMV (Zhang *et al*., 2010, 2013). Indeed, delivery of the BPMV vector into the plant is possible by mechanical inoculation *via* direct DNA rubbing of infectious plasmid DNA, with no need for *in vitro* transcription, *Agrobacterium* transformation or coating to gold particles for biolistic delivery. This efficient, simple and cost‐effective plasmid‐based VIGS vector successfully allowed the functional validation of many genes involved in disease resistance and plant defence in soybean (Cooper *et al*., 2013; Liu *et al*., 2011, 2012; Meyer *et al*., 2009; Pandey *et al*., 2011; Zhang *et al*., 2009, 2012). Recently, we showed that direct DNA rubbing of BPMV‐derived infectious plasmids gives high infection rates in *Phaseolus vulgaris* cv. Black Valentine and induces efficient VIGS in common bean (Pflieger *et al*., 2014).

To test whether VIGS using the ‘one‐step’ BPMV vector was feasible in pea, we first tested the susceptibility to BPMV of six pea genotypes of interest. We also investigated the spatial and temporal infection patterns of BPMV in aerial vegetative and reproductive tissues of these six genotypes. With the goal of adapting the ‘one‐step’ BPMV vector for high‐throughput VIGS studies in pea, we next optimized the conditions for mechanical inoculation of the BPMV vector in pea plants. The efficiency of endogenous gene silencing using the BPMV VIGS vector was investigated by targeting the *PHYTOENE DESATURASE* (*PsPDS*) and the *KORRIGAN‐1* (*PvKOR1*) VIGS reporter genes in aerial parts and roots of cv. Champagne, respectively. Finally, we tested the susceptibility to BPMV of 37 different pea genotypes reflecting the phylogenetic diversity among cultivated peas.

## Results

### Susceptibility to BPMV and spatio‐temporal BPMV infection patterns of aerial tissues of six *P. sativum* cultivars

One inherent property of VIGS is that gene silencing is effective only in genotypes in which the viral vector can multiply and spread systemically throughout the plant. To test the feasibility of BPMV VIGS in pea, we first tested the susceptibility of six pea cultivars (Champagne, FP, PI 180693, AeD99OSW‐50‐2‐5, Isard and 552). These six genotypes were selected because they are the parental lines of recombinant inbred line (RIL) populations used to map genes involved in resistance to *A. euteiches* (Hamon *et al*., 2010, 2013; Pilet‐Nayel *et al*., 2002, 2005), Ascochyta blight (Prioul *et al*., 2004) and genes involved in frost tolerance (Lejeune‐Henaut *et al*., 2008) (Table 1). The susceptibility to BPMV was tested using the BPMV vector expressing the green fluorescent protein GFP (BPMV‐GFP) (Table 2), which was considered as a marker of viral infection (Pflieger *et al*., 2014; Zhang *et al*., 2010). Pea plants were mechanically inoculated using leaf sap obtained from *P. vulgaris*‐infected plants. Indeed, in a previous study, we showed that *P. vulgaris* cv. Black Valentine is highly susceptible to BPMV and that direct rub‐inoculation of BPMV infectious plasmids results in a high infection rate (Pflieger *et al*., 2014). The green fluorescence of GFP was monitored by visual inspection of inoculated pea plants under UV light at different times postinoculation (pi).

**Table 1:** Biological characteristics of the six pea genotypes of interest

Genotype/Accession	Agronomic traits	Organ^a^
Champagne	Resistance to Ascochyta blight and frost tolerance	Leaves
FP	Resistance to Ascochyta blight and frost tolerance	
Isard^b^	Resistance to Ascochyta blight and frost tolerance	
PI 180693	Resistance to *Aphanomyces euteiches*	Roots
552	Resistance to *Aphanomyces euteiches*	
AeD99OSW‐50‐2‐5	Resistance to *Aphanomyces euteiches*	

a Organ in which the agronomic trait is expressed.

b *afila*: mutant phenotype in which leaflets of compound leaves are replaced by tendrils.

**Table 2:** *Bean pod mottle virus* (BPMV)‐derived constructs used in this study

RNA1‐derived plasmid	RNA2‐derived plasmid	Name of the viral vector
pBPMV‐IA‐R1M^a^	pBPMV‐IA‐V1^a^	BPMV‐0
pBPMV‐IA‐R1M^a^	pBPMV‐GFP2^a^	BPMV‐GFP
pBPMV‐IA‐R1M^a^	pBPMV‐IA‐PsPDS‐336bp	BPMV‐PDS
pBPMV‐IA‐R1M^a^	pBPMV‐IA‐PsKOR‐345bp	BPMV‐KOR1a
pBPMV‐IA‐R1M^a^	pBPMV‐IA‐PsKOR‐470bp	BPMV‐KOR1b

a DNA plasmids obtained from C. Zhang (Iowa State University, USA) (Zhang *et al*., 2010). Abbreviations: BPMV: *Bean pod mottle virus*, GFP: green fluorescent protein, PDS: phytoene desaturase, Ps: *Pisum sativum*.

**Figure 1:**
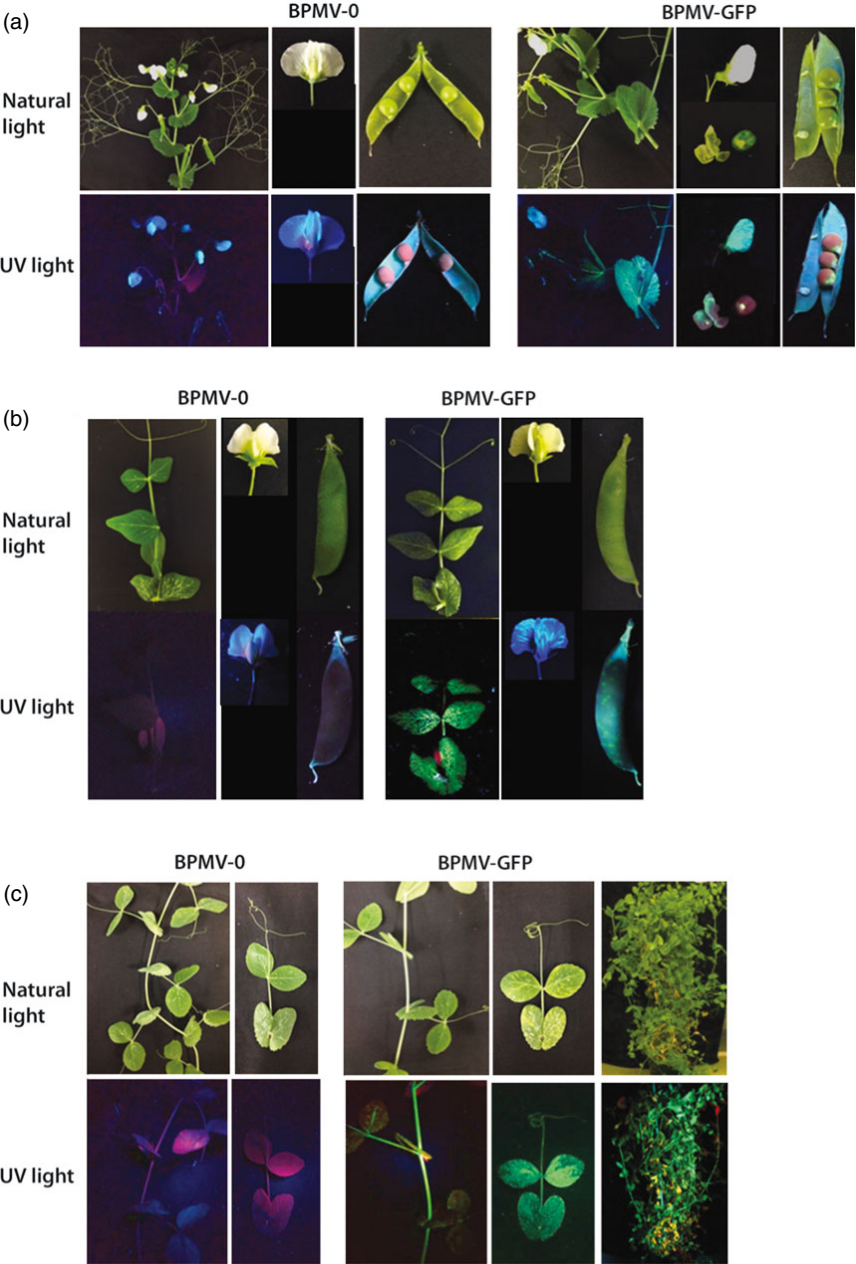
*Bean pod mottle virus* (BPMV)‐induced expression of the green fluorescent protein (GFP) in aerial tissues of *Pisum sativum* cv. Isard, 552 and Champagne. (a) GFP fluorescence in aerial tissues of cv. Isard. Portion of stem, flower and pod infected with BPMV empty vector (BPMV‐0) and *GFP*‐expressing vector (BPMV‐GFP) were photographed at 8 (stem) and 10 weeks postinoculation (wpi) (flower and pod) under natural light (top panel) and UV light (bottom panel). (b) GFP fluorescence in aerial tissues of cv. 552. Upper uninoculated leaf, flower and pod infected with BPMV‐0 and BPMV‐GFP were photographed at 4 wpi, 8 wpi and 10 wpi, respectively, under natural light (top panel) and UV light (bottom panel). (c) GFP fluorescence in aerial tissues of cv. Champagne. Portion of stem and upper uninoculated leaf infected with BPMV‐0 and BPMV‐GFP were photographed at 3 wpi under natural light (top panel) and UV light (bottom panel). Whole plants were photographed at 8 wpi.

For all six pea genotypes, GFP fluorescence was detected after 7 to 14 days pi (dpi) in the inoculated leaves (data not shown) attesting that the BPMV vector has entered into leaf cells and that viral proteins are effectively expressed, including GFP. Fluorescence pattern, in the form of round green spots, corresponded to the primary infection sites. At 4 weeks pi (wpi), GFP fluorescence extended to the upper uninoculated leaves and/or stems in the six genotypes, indicating that the virus is able to multiply in the inoculated leaf and to move systemically (Figures 1 and S1). Significantly, GFP fluorescence was detected as early as 3 wpi in cv. Champagne compared to 4 wpi in all other genotypes. At 8 to 10 wpi, GFP fluorescence was detected in the flowers and/or pods of five genotypes: Isard, 552, PI 180693, AeD99OSW‐50‐2‐5, and FP (Figures 1 and S1). For cv. Champagne, the absence of flower production in our growth conditions explains the lack of data for this genotype. In cv. Isard, GFP fluorescence was visible in the seed coat, whereas no fluorescence was detected in the embryo (Figure 1a). Overall, for the five genotypes Isard, 552, PI 180693, AeD99OSW‐50‐2‐5 and FP, ~30% of the plants inoculated with BPMV‐GFP were systemically infected at 4 wpi. An infection rate of 100% was obtained for cv. Champagne at 4 wpi, and the complete aerial parts were infected at 8 wpi (Figure 1c). Thus, according to our results, we considered that cv. Champagne was the most susceptible genotype to BPMV.

### Optimization of BPMV infection efficiency in *P. sativum*

As efficient VIGS is directly related to efficient viral infection and regarding the low infection rate obtained for five genotypes (Isard, 552, PI 180693, AeD99OSW‐50‐2‐5, and FP), we next optimized the mechanical inoculation procedure. This work was done on two genotypes: Isard and 552. Isard was chosen because it has an *afila* phenotype (leaflets are replaced by tendrils) and because this genotype possesses genes for resistance to Ascochyta blight and tolerance to frost. The genotype 552 has a normal phenotype and presents a high level of resistance to *Aphanomyces euteiches*. As during mechanical inoculation, the virus gets into plant cells by wounding, we tested two intensities of wounding of the upper leaf surface: scarification and abrasion.

As obtained previously, green fluorescence was visible at ~14 dpi in the inoculated leaves of the two genotypes regardless of the inoculation method (data not shown). The scarification inoculation method gave the best inoculation rate for both genotypes Isard and 552 at 4, 5 and 6 wpi (Table 3) and was thus chosen to perform all further mechanical inoculations in pea.

**Table 3:** Optimization of mechanical inoculation of BPMV VIGS vectors in pea

Time of GFP fluorescence observation^a^	4 wpi		5 wpi		6 wpi	
Inoculation	Scarification	Abrasion	Scarification	Abrasion	Scarification	Abrasion
Isard						
Plant 1	+++	−	+++	−	+++	−
Plant 2	++	−	+++	−	+++	−
Plant 3	+	−	+++	−	+++	−
552						
Plant 1	+++	++	+++	++	+++	++
Plant 2	+++	−	+++	−	+++	−
Plant 3	−	−	−	−	−	−

a Observations at 4, 5 and 6 weeks postinoculation (wpi) for two intensities of mechanical inoculation: scarification and abrasion. ‘−’: No fluorescence observed on the aerial parts of the plant; ‘+’, ‘++’, ‘+++’: Fluorescence observed on a quarter, a half or three quarters of the aerial parts of the plant, respectively.

### Virus‐induced gene silencing of *PsPDS* in *P. sativum* cv. Champagne

In the first part of this work, we demonstrated that the six pea genotypes of interest were susceptible to BPMV and that VIGS is thus theoretically feasible in these genotypes. However, to evaluate the efficiency of BPMV VIGS, we focused on the most susceptible pea genotype: cv. Champagne. We constructed a BPMV‐PDS VIGS vector containing a 336‐bp insertion derived from the 3′‐end of the *PsPDS* ORF of cv. Champagne. *PDS* is routinely used as a reporter gene in VIGS assays in plants as silencing of this gene causes chlorophyll degradation resulting in a typical photobleached phenotype in emerging leaves (Kumagai *et al*., 1995). To produce a BPMV‐PDS inoculum suitable for cv. Champagne inoculation, we first inoculated a mix of infectious plasmids corresponding to the BPMV‐PDS construct (Table 2) in *P. vulgaris* cv. Black Valentine. Three wpi, typical photobleaching was observed on young uninoculated leaves of ~60% of the inoculated cv. Black Valentine plants (Figure S2). This observation confirms that the BPMV‐PDS vector is functional and reveals that heterologous VIGS is induced in *P. vulgaris*. Indeed, a sequence alignment showed that the *PDS* fragment from pea shares 91% nucleic acid identity with its homologue in common bean (data not shown).

**Figure 2:**
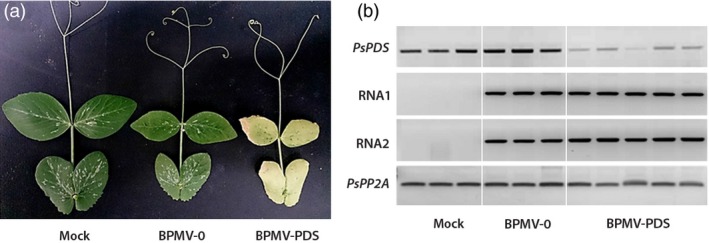
Silencing of *PsPDS* in *P. sativum* cv. Champagne using the VIGS vector BPMV‐PDS (a) Systemic leaves from plants infected with mock buffer, BPMV‐0 and BPMV‐PDS were photographed at 6 weeks postinoculation (wpi). (b) Semi‐quantitative RT‐PCR of *PsPDS*, BPMV RNA1 and RNA2 in systemic leaves of plants inoculated with mock, BPMV‐0 and BPMV‐PDS. Phosphoprotein phosphatase 2A (*PsPP2A*) was used as an internal control. Total RNA was extracted at 6 wpi from systemic leaves of three (Mock, BPMV‐0) or five (BPMV‐PDS) different plants of cv. Champagne.

White photobleached leaves of *P. vulgaris* were used as inoculum to produce leaf sap. Mechanical inoculation of pea cv. Champagne was performed using the scarification method described above. At 6 wpi, plants inoculated with BPMV‐PDS displayed a clear photobleached phenotype with completely white newly emerging leaves, unlike plants infected with the empty BPMV‐0 vector or mock buffer (Figure 2a). To confirm that the photobleached phenotype described above correlated with reduced endogenous levels of *PsPDS*, semi‐quantitative RT‐PCR was carried out on uninoculated leaves from each of the three treatment groups (Figure 2b). To test whether the phenotype observed in treated plants could be due to the presence of the viral vectors, the presence of BPMV RNA1 and RNA2 transcripts was also determined by RT‐PCR (Figure 2b, 2 middle gels). As expected, samples from the mock‐treated plants did not show viral RNA1 and RNA2 unlike BPMV‐0‐ and BPMV‐PDS‐inoculated plants (Figure 2b). BPMV‐0‐inoculated plants showed expression levels of *PsPDS* similar to that of mock‐treated plants, suggesting that the viral treatment does not interfere with *PsPDS* expression (Figure 2b). In samples from the BPMV‐PDS treated plants, there was a strong down‐regulation of *PsPDS* (relative to *PsPP2A*) (Figure 2b).

### BPMV infection of roots of six *P. sativum* cultivars

**Figure 3:**
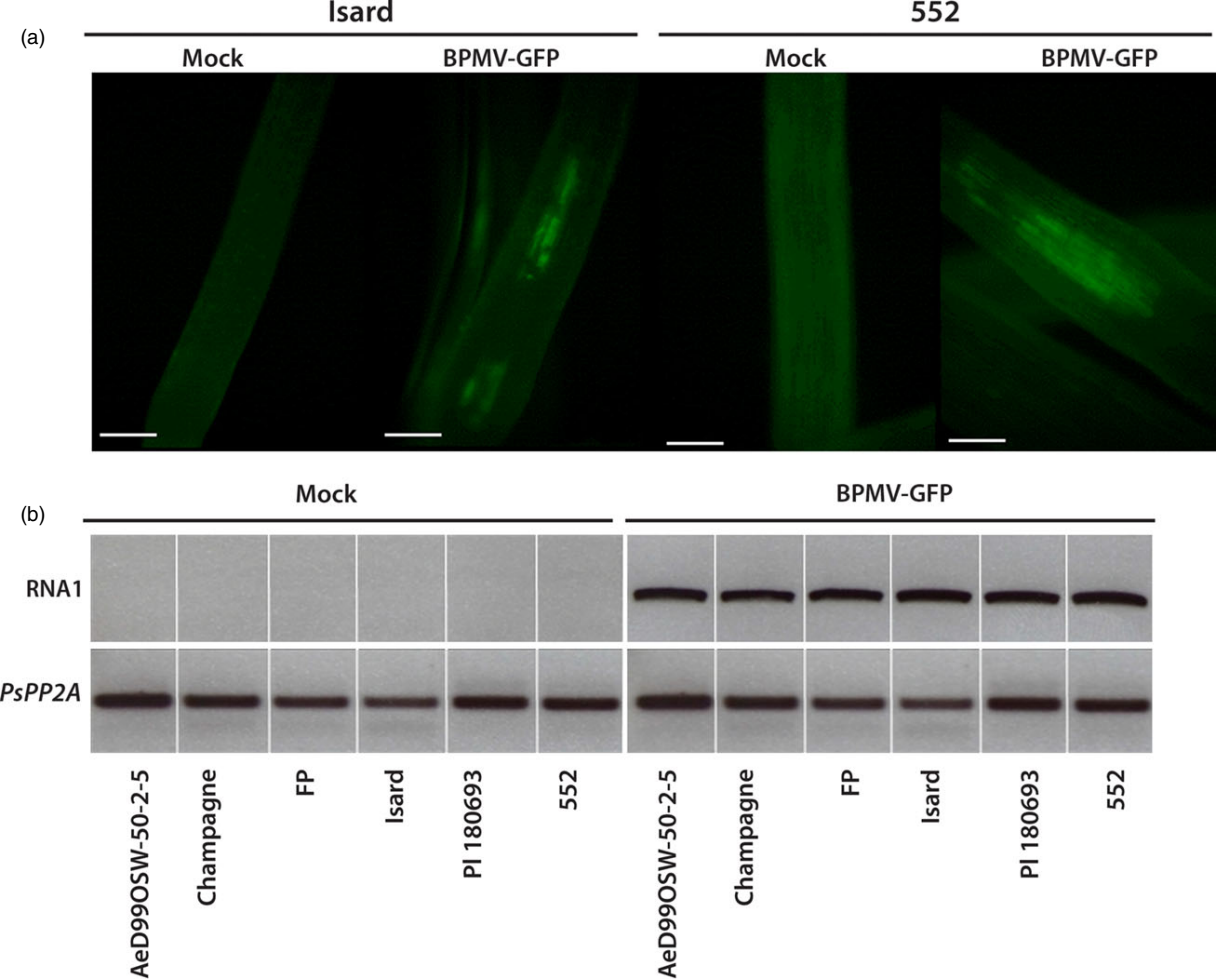
*Bean pod mottle virus* (BPMV)‐induced expression of the green fluorescent protein (GFP) gene in roots of *P. sativum* genotypes of interest. (a) Epifluorescence microscope observation of GFP fluorescence in young lateral roots of cv. Isard (at 6 weeks postinoculation (wpi)) and cv. 552 (at 5 wpi) inoculated with BPMV‐GFP. Bar = 250 microns. (b) Semi‐quantitative RT‐PCR of BPMV RNA1 in roots of plants inoculated with mock and BPMV‐GFP. The *PsPP2A* (phosphoprotein phosphatase 2A) gene was used as an internal control. Total RNA was extracted at 4 wpi from roots of each genotype.

As one of our aims in developing BPMV‐derived VIGS for pea is to identify genes involved in resistance to the root pathogen *A. euteiches*, we next investigated whether BPMV could be a suitable VIGS vector for silencing endogenes expressed in roots. To examine whether BPMV infects roots, we first tested the presence of GFP fluorescence in the root systems of genotypes Isard and 552 at 6 and 5 wpi, respectively. When young lateral roots were observed with an epifluorescence microscope, GFP fluorescence was detected for the two genotypes Isard and 552 (Figure 3a). As an alternative to microscope observations, we tested the presence of BPMV‐GFP in the roots of all six genotypes using semi‐quantitative RT‐PCR analyses. Total RNAs were extracted from roots of BPMV‐GFP‐infected plants and control plants at 4 wpi. PCR using primers specific to BPMV RNA1 showed the presence of viral RNAs in the roots of BPMV‐GFP‐infected plants for all six genotypes, whereas no viral RNAs were detected in control plants as expected (Figure 3b). Thus, the results confirmed the presence of BPMV viral RNAs in roots of the six genotypes of interest.

### Virus‐induced gene silencing of *PsKOR1* in *P. sativum* cv. Champagne

To test BPMV VIGS in roots, we constructed a BPMV‐KOR1 vector containing a fragment of the *KORRIGAN‐1* gene of *P. sativum* (*PsKOR1*). In *Arabidopsis thaliana*, the *KOR1* gene encodes an endo‐1,4‐β‐D‐glucanase involved in cellulose synthesis (Sato *et al*., 2001). Mutants carrying T‐DNA insertions in the putative promoter region display an extreme dwarf phenotype with severe stunting of both stem and roots (Nicol *et al*., 1998). Silencing of the *KOR1* gene in *P. sativum* using the PEBV VIGS vector resulted in a reduced plant height and a shorter root length (Constantin *et al*., 2004). We constructed two BPMV‐KOR1 vectors. The first vector (so‐called BPMV‐KOR1a) contains a 345‐bp length fragment derived from the 3′‐end of the *PsKOR1* ORF of cv. Champagne (Table 2). Indeed, according to Zhang *et al*. (2010, 2013), silencing efficiency using the ‘one‐step’ BPMV vector is generally higher for the 3′ region of the target gene ORF and for a fragment size of ~300–400 bp. The second vector (so‐called BPMV‐KOR1b) contains a 470‐bp length fragment homologous to the 470‐bp fragment used by Constantin *et al*. (2004) in the PEBV vector (Table 2). The two *KOR1* fragments (345 bp and 470 bp) overlap by 181 bp on the *PsKOR1* mRNA. We first inoculated the two BPMV‐KOR1 constructs in *P. vulgaris* cv. Black Valentine by direct DNA rubbing of a mix of two infectious plasmids (Table 2). Three wpi, typical viral symptoms were observed on uninoculated systemic leaves of ~60% of the inoculated cv. Black Valentine plants (Figure S2), but infected plants did not display a reduced plant height (data not shown). This may be due to the low nucleic acid identity (<50%) between the *KOR1* fragments of the *PsKOR1* gene of pea and *PvKOR1* expressed in roots of common bean.

**Figure 4:**
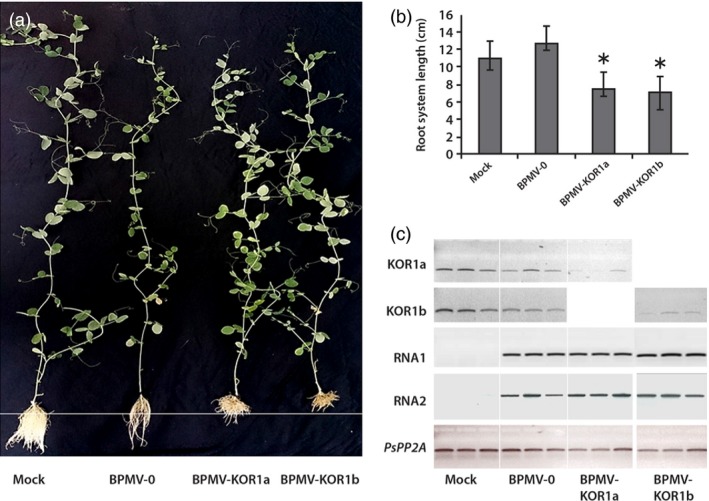
Silencing of *KOR1* in *P. sativum* cv. Champagne using the VIGS vectors BPMV‐KOR1a and BPMV‐KOR1b. (a) Plants inoculated with mock, BPMV‐0, BPMV‐KOR1a and BPMV‐KOR1b were photographed under natural light at 4 weeks postinoculation (wpi). (b) Root system lengths (in centimetre) of plants inoculated with mock, BPMV‐0, BPMV‐KOR1a and BPMV‐KOR1b measured at 4 wpi. Data are the mean of two samples for mock‐treated plants, three samples for BPMV‐0‐inoculated plants and seven samples for BPMV‐KOR1a‐ and BPMV‐KOR1b‐inoculated plants ± SE. Mean values significantly different from the BPMV‐0‐inoculated plants are indicated by an asterisk as determined by paired *t*‐test (*P* < 0.01) (c) Semi‐quantitative RT‐PCR of *PsKOR1*, BPMV RNA1 and RNA2 in roots of plants inoculated with mock, BPMV‐0, BPMV‐KOR1a and BPMV‐KOR1b. The *PsPP2A* (phosphoprotein phosphatase 2A) gene was used as an internal control. Total RNA was extracted at 4 wpi from root systems of three different plants for each construct.

Infected leaves of *P. vulgaris* were used as inoculum to produce leaf sap for mechanical inoculation of pea cv. Champagne. Inoculated plants were put in hydroponic culture. At 4 wpi, root length of each plant was measured and representative plants were photographed (Figure 4a). Roots of plants inoculated with BPMV‐KOR1a or BPMV‐KOR1b were significantly shorter than roots of plants inoculated with BPMV‐0 (Figure 4a and b) supporting the hypothesis that silencing of *PsKOR1* had taken place. To correlate this root dwarf phenotype with the transcript extinction of *PsKOR1*, we performed semi‐quantitative RT‐PCR in roots at 4 wpi. Total RNA was extracted from plants inoculated with mock, BPMV‐0, BPMV‐KOR1a and BPMV‐KOR1b. To test whether the phenotype observed in treated plants could be due to the presence of the viral vectors, the presence of BPMV RNA1 and RNA2 transcripts was also determined by RT‐PCR (Figure 4c). As expected, no viral RNAs were detected in the roots of the mock‐treated plants, unlike in all other samples (Figure 4c). Expression levels of *PsKOR1* were similar in roots of BPMV‐0‐inoculated plants compared to mock‐treated plants suggesting that the viral infection does not affect the expression level of *PsKOR1* (Figure 4c). Transcripts levels of *PsKOR1* were significantly decreased in samples from roots of plants inoculated with either BPMV‐KOR1a or BPMV‐KOR1b, relative to the control plants (mock and BPMV‐0) and relatively to *PsPP2A* (Figure 4c). No significant difference was detected between BPMV‐KOR1a and BPMV‐KOR1b samples.

### Screening for susceptibility to BPMV of a *P. sativum* panel of diversity

In this work, we demonstrated that six pea genotypes (Champagne, FP, PI 180693, AeD99OSW‐50‐2‐5, Isard and 552) were all susceptible to BPMV and that efficient VIGS was induced using the ‘one‐step’ BPMV vector in leaves and roots of cv. Champagne. To further extend the application of VIGS using the ‘one‐step’ BPMV vector in *P. sativum* and as VIGS technology can be used only in susceptible genotypes, we carried out a screening experiment using 37 different pea genotypes reflecting the diversity of cultivated peas (Table 4). Three plants of each genotype were tested in at least two independent experiments. Pea plantlets were inoculated at the two‐leaf stage using the high‐intensity inoculation method and leaf sap obtained from BPMV‐GFP‐infected leaves of *P. vulgaris* cv. Black Valentine. The presence of GFP fluorescence was scored under UV light at 3 and 4 wpi in inoculated leaves and uninoculated leaves, respectively, for the 37 pea genotypes, and results were synthesized in Table 4. Twenty‐eight genotypes were infected systemically by BPMV‐GFP (noted ‘YES’ in Table 4). Two genotypes (cv. Enduro and James) displayed GFP fluorescence in the inoculated leaves only (noted ‘YES only in IL’ in Table 4), indicating that systemic movement of BPMV‐GFP was restricted. Seven genotypes displayed no GFP fluorescence in either inoculated or uninoculated leaves (noted ‘NO’ in Table 4).

**Table 4:** Susceptibility to BPMV of *Pisum sativum* genotypes tested by mechanical inoculation with BPMV‐GFP

N°	*P. sativum* genotype	Presence of GFP fluorescence indicative of BPMV infection^a^				Susceptibility to BPMV
		3 wpi		4 wpi		
		Inoculated leaves	Uninoculated leaves	Inoculated leaves	Uninoculated leaves	
1	Champagne	+	+	+	+	YES
2	CE101 = FP	+	+	+	+	YES
3	Isard	+	+	+	+	YES
4	552	+	+	+	+	YES
5	PI180693	+	+	+	+	YES
6	AeD99OSW‐50‐2‐5	+	+	+	+	YES
1	Yangwan G1503	−	−	−	−	NO
2	Merveille d'Etampes	+	−	−	+	YES
3	Livioletta	+	+	−	+	YES
4	Ballet	−	−	−	−	NO
5	Petit Provençal	+	+	+	+	YES
6	Pisum sativum‐AFGHANISTAN JI86	+	+	+	+	YES
7	Vavilov‐D261	+	+	+	+	YES
8	Capsicum	−	+	+	+	YES
9	Shrat	−	−	−	−	NO
10	Afghanistan Asiaticum	−	−	−	−	NO
11	WNC 23Z SPP ARVENSE 1809	+	+	+	+	YES
12	Côte d'Or	+	+	+	+	YES
13	336/11	+	+	+	+	YES
14	DP	+	+	+	+	YES
15	AeD9904‐7	+	−	+	+	YES
16	Eden	+	+	+	+	YES
17	Cheyenne	−	−	−	−	NO
18	Geronimo	+	+	+	+	YES
19	Baccarat	−	−	+	+	YES
20	Glacier	+	+	+	+	YES
21	Austrian Winter	+	+	+	+	YES
22	Cameor	−	−	−	−	NO
23	Kayanne	+	+	+	+	YES
24	MN 314	−	−	+	+	YES
25	90‐2079	+	−	+	+	YES
26	0‐2073	+	+	+	+	YES
27	90‐2131	+	−	+	+	YES
28	RIL 847‐50	+	−	+	+	YES
29	E11	+	+	+	+	YES
30	Ethiopia‐32 CGN 3255	+	−	+	+	YES
31	Spartanets	+	+	+	+	YES
32	Terese	+	−	+	+	YES
33	AeD99OSW‐45‐8‐7	+	+	+	+	YES
34	Enduro	+	−	+	−	YES only in IL
35	Torsdag	−	−	−	−	NO
36	James	+	−	+	−	YES only in IL
37	China	+	+	+	+	YES

a ‘−’: no visible GFP fluorescence; ‘+’: visible GFP fluorescence; IL: inoculated leaves.

## Discussion

*Pisum sativum* is an economically important crop especially in Europe. The genome of pea is about to be completely sequenced by the International Pea Sequencing Project consortium (Alves‐Carvalho *et al*., 2015; McGee, 2013). To relate the identified genes with important agronomic traits, functional genomics tool are needed at the genome‐wide level. In pea, as in other legumes, stable genetic transformation is relatively inefficient (Somers *et al*., 2003; Svabova *et al*., 2005) and VIGS has become an important reverse genetics tool for functional genomics in these species (Pflieger *et al*., 2013). In the present study, we investigated whether BPMV‐based vector, the most widely used VIGS vector in legumes, could be effective for silencing of endogenous genes and for foreign gene expression in pea.

In pea, a VIGS vector was originally developed from PEBV that induced an efficient and reliable gene silencing (Constantin *et al*., 2004). *P. sativum* cv. Scout inoculated with PEBV‐PDS developed the characteristic photo‐bleached phenotype on 95% of the upper leaves at 42 dpi (Constantin *et al*., 2004). Using the BPMV VIGS vector, we observed comparable intensity and timing of silencing induction for *PsPDS* as leaf photobleaching in cv. Champagne was observed at 6 wpi and this phenotype was associated with a reduction in *PDS* transcripts. In roots, silencing of *PsKOR1* gene was shown to occur at 4 wpi both in cv. Scout (Constantin *et al*., 2004) and in cv. Champagne (this study). In conclusion, the one‐step BPMV VIGS vector is as effective as the PEBV VIGS vector for silencing of endogenes in pea.

One critical step when making VIGS is the delivery of the recombinant viral vector into plant cells to initiate viral infection (*i. e*. primary inoculation). Delivery can be achieved by various techniques such as *Agrobacterium*‐mediated infiltration (agro‐inoculation), mechanical inoculation (generally by leaf rubbing) of *in vitro*‐transcribed RNA or biolistic delivery of infectious plasmid DNA (Burch‐Smith *et al*., 2004; Robertson, 2004). In the case of PEBV, initial delivery is achieved by agro‐inoculation because PEBV is not transmitted mechanically (Constantin *et al*., 2004). In the aim of making high‐throughput VIGS studies, this technique may become laborious and time‐consuming because of the step of *Agrobacterium* transformation (Pflieger *et al*., 2013). On the contrary, BPMV is a ‘one‐step’ VIGS vector delivered into plants via direct DNA rubbing of infectious plasmid DNAs which is a rapid, simple and cost‐effective procedure for making low‐ to high‐throughput genomic studies (Pflieger *et al*., 2013; Zhang *et al*., 2010).

As VIGS efficiency is correlated with the virus titre in the infected plants (Senthil‐Kumar and Mysore, 2011), we performed all primary inoculations of infectious plasmid DNAs in *P. vulgaris* cv. Black Valentine because this genotype is highly susceptible to BPMV (Pflieger *et al*., 2014; Zhang *et al*., 2010). Systemically infected leaves of cv. Black Valentine are trifoliate leaves, and each leaflet constitutes a highly efficient inoculum for making secondary inoculations. This is an advantage compared to pea leaflet that are quite smaller and that would produce less inoculum material not suitable for high‐throughput genomic studies. In addition, we demonstrated that the secondary infection rates in pea ranges from 100% (in cv. Champagne) to 30% (cv. FP, PI 180693, AeD99OSW‐50‐2‐5, Isard and 552). Thus, to optimize chances to have a successful infection of pea genotypes, we recommend to use a highly efficient viral inoculum produced in *P. vulgaris* cv. Black Valentine.

Our results showed that BPMV infection in pea only generates mild mosaics (Figures 1 and 4, S1), therefore not interfering with the phenotype associated with silencing of the gene of interest. This feature is of great importance when making VIGS studies because viral infection may alter defence responses, development and morphology of the silenced plant. Thus, it is recommended if possible to work with mild viral strains. This is the case for the ‘one‐step’ BPMV VIGS vector, which is derived from the IA‐Di1 isolate of BPMV inducing very mild visual symptoms on infected soya bean plants. In common bean, we showed that although viral symptoms were present (mottling and distorted leaves), they do not interfere with the silencing phenotypes (Pflieger *et al*., 2014). Nevertheless, it is important to include a positive control (i.e. plant infected with an empty VIGS vector) in all VIGS assays to check the effect of viral infection on the silenced plant.

The ideal size of the foreign sequence inserted into the viral vector for VIGS induction ranges between an upper size limit allowing the virus to replicate and to move systemically and a lower size limit allowing efficient VIGS induction (Burch‐Smith *et al*., 2004; Robertson, 2004). These two parameters depend on the chosen virus and on the host plant genotype. Moreover, orientation of the foreign sequence inserted into the viral vector and the position of the target region inside the target gene are critical for silencing intensity. With the ‘one‐step’ BPMV VIGS vector, fragments in the range of 100–500 nucleotides, inserted in antisense orientation and targeting the ORF 3′‐end of the target gene, are recommended to ensure efficient silencing (Juvale *et al*., 2012; Pandey *et al*., 2011; Zhang *et al*., 2013). Based on these parameters, we used an insert of 336 and 345 nucleotides selected in the ORF 3′‐region of *PsPDS* and *PsKOR1* genes, respectively, to construct the two BPMV vectors BPMV‐PDS and BPMV‐KOR1a. For *PsKOR1*, we also tested BPMV‐KOR1b containing the same 470‐nt fragment as previously used with the PEBV vector (Constantin *et al*., 2004). Although this insert does not comply with the choice of the ORF 3′‐end region, the observed results showed no significant difference between silenced plants with BPMV‐KOR1a and BPMV‐KOR1b (Figure 4c). This can be explained by the fact that the 470‐nt fragment of BPMV‐KOR1b covers a portion of the 3′ region because the whole *PsKOR1* ORF has a length of 688 nt.

One limitation of VIGS is that it is possible only in genotypes where the viral vector can spread systemically. For a given species, different genotypes might react differently to viral infection, from total resistance due to specific resistance genes to various levels of susceptibility. This has been well documented for the PEBV‐derived vector in *Medicago truncatula* and *Lathyrus odorata* (Grønlund *et al*., 2008). Consequently, it is essential to test the susceptibility to BPMV of each genotype of interest. To our knowledge, the BPMV vector has never been tested on pea genotypes. In a first step, we analysed the susceptibility to BPMV of six pea genotypes of interest and found that all six were susceptible to BPMV (Figures 1 and 3, S1). In these six genotypes, BPMV spread systemically throughout the whole plant (Figures 1and 3, S1). Following BPMV‐GFP infection, fluorescence was detected in primary leaves, trifoliate leaves, stems, roots, floral buds, flowers, pods and seed coats (Figures 1and 3, S1). As VIGS is more effective only in tissues infected by the viral vector, it should therefore be possible to induce gene silencing in all these organs. In a second step, we investigated 37 additional pea genotypes and found that 28 were susceptible to BPMV, two genotypes displayed fluorescence only in the inoculated leaf, and only 7 seemed resistant (Table 4). Unfortunately, the genotype Cameor, the genome of which is currently being sequenced (Alves‐Carvalho *et al*., 2015; McGee, 2013), was found to be part of the 7 resistant genotypes. To affirm that these 7 genotypes are completely resistant to BPMV, infection with the empty BPMV vector should be tested because BPMV‐GFP may be less efficient to infect these genotypes. Nevertheless, the BPMV vector is usable in the 6 + 28 pea genotypes provided further investigation to improve infection rates, viral spread and VIGS efficiency.

Implementation of BPMV VIGS in pea further supports the use of this VIGS vector in other important legumes for which stable transgenic techniques are not yet available. The extent to which the ‘one‐step’ BPMV vector can be used in other legume species (except soybean, common bean, and pea) is unknown, and it would be interesting to test the susceptibility to BPMV in species such as *Cajanus cajan*,* Cicer arietinum*,* Vigna radiata* whose genomes have recently been sequenced (Kang *et al*., 2014; Varshney *et al*., 2012, 2013), as well as in the model legume *Medicago truncatula* (Young *et al*., 2011).

Plant virus‐based vectors are also valuable tools for heterologous protein expression in plants. These vectors are fast‐acting, cost‐efficient and high yield, and significantly, they can be used in a variety of genetic backgrounds provided that the selected genotype is susceptible to the viral vector. Here, we show that the BPMV vector is a suitable virus‐based system that is effective for stable expression of a foreign protein in pea. We found that a recombinant BPMV‐GFP vector gives an intense GFP expression in a large range of vegetative and reproductive organs in several susceptible pea genotypes (Figures 1 and S1). Further investigations are needed to determine the maximal insert size compatible with virus infectivity and systemic movement for a given genotype. Previous studies have estimated the BPMV RNA2 vector capacity for foreign gene insertion to be between 1.4 and 1.8 kb (Zhang and Ghabrial, 2006; Zhang *et al*., 2010). Although the average length of a plant gene is ~3 kb, it could be possible to express short proteins such as viral or fungal effectors involved in plant immune responses.

In this study, we found that seven pea genotypes were resistant to BPMV‐GFP. Resistant genotypes have also been identified previously in common bean (Mozzoni *et al*., 2009; Pflieger *et al*., 2014). To our knowledge, no resistance to BPMV has been reported so far in soybean (Mozzoni *et al*., 2009; Wang *et al*., 2005; Zheng *et al*., 2005). This result suggests (i) that pea has an *R* gene conferring resistance to BPMV that could be an orthologue of *R‐BPMV* (the *R* gene present in common bean cv. BAT93) (Pflieger *et al*., 2014) or (ii) that pea may have one or several other different *R* genes conferring resistance to BPMV. As the resistant pea genotypes do not belong to a single group of diversity (data not shown), we favour the latter hypothesis. However, genetic analysis will be necessary to confirm whether one or several *R* genes are involved in BPMV resistance in *P. sativum*.

## Experimental procedures

### Plant material and growth conditions

Seeds of *Phaseolus vulgaris* cv. Black Valentine used for primary inoculations in this study were obtained from multiplications performed at IPS2, Orsay (France). All seeds of *Pisum sativum* genotypes were obtained from multiplications performed at INRA Rennes (France) and INRA Dijon (France). The thirty‐seven genotypes used for the screening for susceptibility to BPMV belong to a panel of diversity of cultivated *P. sativum* established at INRA Dijon (unpublished results). Seedlings of common bean and pea genotypes were cultivated as described previously (Pflieger *et al*., 2014, 2015). Before inoculation, pea seedlings for *PsKOR1* silencing assays were placed individually in hydroponic culture using a nutrient solution as substrate (Pflieger *et al*., 2015). At 14 dpi, roots were cut back to approximately 3 cm and root growth was allowed to re‐initiate as described by Constantin *et al*. (2004) in PEBV VIGS experiments.

### Viral vectors

Names of the BPMV constructs used in this study and the corresponding RNA1‐ and RNA2‐derived plasmids are listed in Table 2. The pBPMV‐IA‐R1M, pBPMV‐IA‐V1 and pBPMV‐GFP2 DNA plasmids were kindly supplied by C. Zhang from Iowa State University, USA (Zhang *et al*., 2010). All other RNA2‐derived plasmids were constructed in our laboratory by insertion of *P. sativum* target gene fragments in the *Bam*H1 restriction site of pBPMV‐IA‐V1. Sequences of 1077 bp corresponding to *PDS* mRNA of *P. sativum* (GenBank accession AJ621573.1, cv. Scout) and 688 bp corresponding to *KOR1* mRNA (GenBank accession AJ621355.1, cv. Scout) were used as matrix to determine the PCR primers using the software Primer3 (Untergasser *et al*., 2012). Target gene fragments were first amplified by PCR using specific primers (Table S1), *P. sativum* cDNAs as template and a high‐fidelity polymerase (Advantage^®^‐HF2, Clontech, Mountain View, CA, USA). DNA plasmids pBPMV‐IA‐PDSrev336pb, pBPMV‐IA‐KORrev‐345pb and pBPMV‐IA‐KORrev‐470pb (Table 2) were constructed by cloning cohesive‐end fragments of 336 bp of the *PsPDS gene*, and 345 and 470 bp of the *PsKORRIGAN1* (*KOR1*) gene, respectively, into *Bam*H1‐digested and dephosphorylated pBPMV‐IA‐V1. The orientation of the cloned inserts was determined by PCR using a combination of vector‐specific and fragment‐specific primers (Table S1). Clones thought to contain inserted fragments in the antisense orientation were sequenced (GATC, Konstanz, Germany) to confirm their identity, and a maxi‐preparation of plasmid DNA was performed using the ‘QIAGEN Plasmid Purification’ (Hilden, Germany) kit.

### Viral inoculation procedure

Primary inoculations with BPMV‐derived infectious plasmids were performed in common bean cv. Black Valentine as described previously (Pflieger *et al*., 2014, 2015). For secondary inoculations of pea genotypes, an infected leaf of *P. vulgaris* cv. Black Valentine harvested at 4 to 5 wpi (fresh leaf or frozen leaf conserved at −80 °C) was ground in a mortar with mock buffer to make leaf sap. Mechanical inoculations were performed by rubbing of the leaflets and stipules of two leaves of pea plantlets at the two‐leaf stage of development (approximately 10‐ to 12‐day‐old plantlets in our growth conditions). Plants inoculated with either inoculation buffer without virus (Mock) or empty BPMV vector (BPMV‐WT) were used as controls. After viral inoculation, the potted plants or hydroponic‐cultured plants were placed in a growth room at 19 °C under a 16‐h light/8‐h dark cycle under a humidity of 70% and watered with nutrient solution (Pflieger *et al*., 2015).

### Optimization of conditions for mechanical inoculation of leaf sap in *P. sativum*

To increase the infection rate in pea, two intensities of mechanical inoculation were tested on the two younger leaves and stipules of 10‐ to 12‐day‐old plants of cv. Isard and 552: abrasion and scarification. For abrasion, the leaf was dusted with carborundum and rubbed with miracloth soaked with leaf sap. For scarification, a scalpel blade was used to make superficial incisions in the waxy layer and in the upper epidermis of the leaf. Depending on the size of the leaflet surface, four to six parallel incisions were made on one leaflet. The wounded upper leaf surface was then rubbed as in the abrasion inoculation method. Six seedlings of both cv. Isard and 552 were inoculated with BPMV‐GFP, with three plants per inoculation method. Inoculum consisted of leaf sap derived from *P. vulgaris* cv. Black Valentine plants infected with BPMV‐GFP. After inoculation, the GFP fluorescence was scored in the inoculation leaf at 14 dpi and in the upper systemic leaves at 4 and 5 wpi using a quantitative scale of notation.

### GFP imaging

GFP expression in aerial organs and roots was examined using a UV lamp (High‐intensity 100‐Watt long‐wave UV lamp, UVP, USA) and photographed using a Canon PowerShot A2300 digital camera. Microscopic observations of roots were made using an Axioskop microscope (Zeiss, Germany) and photographed using a RTKE camera (SPOT imaging, Sterling Heights, MI, USA).

### Expression analysis by semi‐quantitative RT‐PCR

Total RNA and cDNA syntheses were performed as described in Richard *et al*. (2014). Primer sequences are listed in Table S1. Primers for RT‐PCR analyses of *PsPDS* and *PsKOR1* genes were designed to anneal outside the region targeted for silencing so that only the endogenous gene was probed. Primers PsPP2A‐fwd and PsPP2A‐rev were used to amplify *PsPP2A* as an internal constitutively expressed mRNA control (Die *et al*., 2010). After 25 cycles, PCR products were analysed using analytical agarose–ethidium bromide electrophoresis.

### Statistical analysis

Statistically significant differences were analysed by Student's *t*‐test. Significance was set at *P* < 0.01.

## Supporting information


**Figure S1** *Bean pod mottle virus* (BPMV)‐induced expression of the green fluorescent protein (GFP) in aerial tissues of *Pisum sativum* cv. PI 180693, AeD99OSW‐50‐2‐5, and FP.
**Figure S2 **Viral symptoms and silencing phenotypes induced by BPMV VIGS vectors in primary‐inoculated plants of *P. vulgaris* cv. Black Valentine.
**Table S1** PCR primers used for construction of the BPMV VIGS vectors and RT‐PCR analyses.
