# Supplementary material for: Bean pod mottle virus: a new powerful tool for functional genomics studies in Pisum sativum
Source: Plant Biotechnol J. 2016 Feb 20;14(8):1777–87. doi: 10.1111/pbi.12537 (PMC5067550; doi:10.1111/pbi.12537)
Supplement: Supplementary file 1 — Figure S1 Bean pod mottle virus (BPMV)‐induced expression of the green fluorescent protein (GFP) in aerial tissues of Pisum sativum cv. PI 180693, AeD99OSW‐50‐2‐5, and FP. Figure S2 Viral symptoms and silencing phenotypes induced by BPMV VIGS vectors in primary‐inoculated plants of P. vulgaris cv. Black Valentine. Table S1 PCR primers used for construction of the BPMV VIGS vectors and RT‐PCR analyses. [file PBI-14-1777-s001.docx]

**Supporting information**

**Figure S1**

**
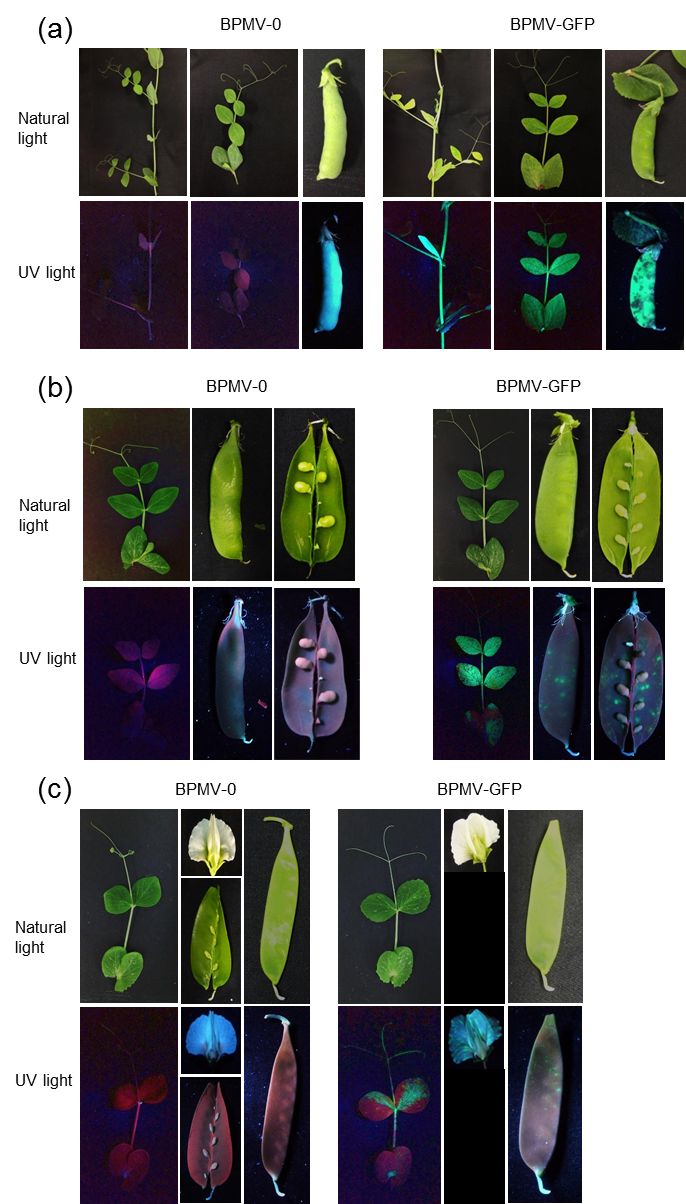
**

**Figure S1** *Bean pod mottle virus* (BPMV)-induced expression of the green fluorescent protein (GFP) in aerial tissues of *Pisum sativum* cv. PI 180693, AeD99OSW-50-2-5, and FP. (a) GFP fluorescence in aerial tissues of cv. PI 180693. Portion of stem, upper uninoculated leaf, and pod infected with BPMV empty vector (BPMV-0) and *GFP*-expressing vector (BPMV-GFP) were photographed at 4 weeks post-inoculation (wpi) and 10 wpi (pods), under natural light (top panel) and UV light (bottom panel). (b) GFP fluorescence in aerial tissues of cv. AeD99OSW-50-2-5. Upper uninoculated leaf and pod infected with BPMV-0 and BPMV-GFP were photographed at 4 wpi and 10 wpi (pods), under natural light (top panel) and UV light (bottom panel). (c) GFP fluorescence in aerial tissues of cv. FP. Upper uninoculated leaf, flower, and pod infected with BPMV-0 and BPMV-GFP were photographed at 8 wpi, and 10 wpi (pods), under natural light (top panel) and UV light (bottom panel).

**Figure S2**

**
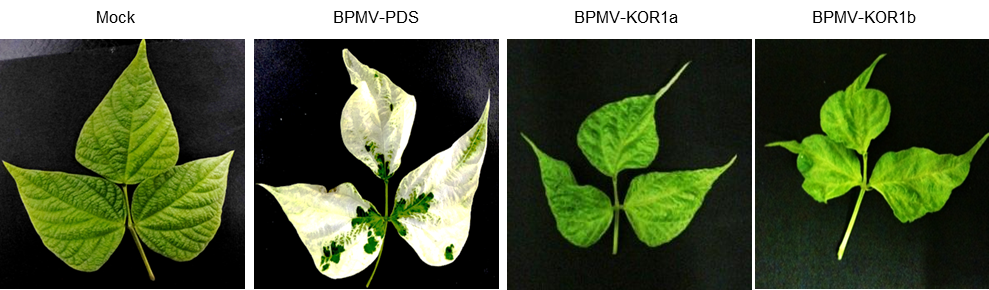
**

**Figure S2**Viral symptoms and silencing phenotypes induced by BPMV VIGS vectors in primary-inoculated plants of *P. vulgaris* cv. Black Valentine. Systemic leaves from *P. vulgaris* cv. Black Valentine plants infected either with mock buffer or with a DNA plasmid mix corresponding to BPMV-PDS, BPMV-KOR1a and BPMV-KOR1b, were photographed at 3 weeks post-inoculation.

**Table S1** PCR primers used for construction of the BPMV VIGS vectors and RT-PCR analyses

| Name of the primer | DNA sequence of the primer (5’-3’) | Size of PCR product (bp) |
| --- | --- | --- |
| PsPDS-336-fwd  PsPDS-336-rev | CGGGATCCAGCAGAAGCCCCCTTCTGAG  CGGGATCCttgttttgtgtaatctcctg | 336 |
| PsKOR1-345-fwd  PsKOR1-345-rev | CGGGATCCGGGAAGAATCCACGGAAAAT  CGGGATCCCATTGGTGGAACAGCAGAGA | 345 |
| PsKOR1-470-fwd  PsKOR1-470-rev | CGGGATCCTGGGTATCCATATGAAGAA  CGGGATCCCAGCAACCATGGCACCAA | 470 |
| RNA1bis-fwd  RNA1bis-rev | GAGATGGTGAAGAAATGTCACTAA  AAGCAGCGCGTCTTGGGTTTATCA | 160 |
| RNA2-fwd  RNA2-rev | ATACCCCTAATGGCACAGGA  GGAAATGTAACCACCCGAAT | 269 |
| RNA2-*Bam*H1-fwd  RNA2-*Bam*H1-rev | TGACAATCCCAAACAGTCTACAG  AGCATACTCAACGAGAGGGTCA | 234 |
| PsPDS-fwd  PsPDS-rev | GCCAGCTATTCTTGGTGGA  Cgcccaatgactgaatatga | 287 |
| KOR1a-fwd  KOR1a-rev | GGTTGTTCTTGAGTCCTGGGTA  GGTAGCCAAAAAGGCTGCAT | 191 |
| KOR1b-fwd  KOR1b-rev | GACAGGCACGACGGTTTC  TGTTGGAAACATTGGTGGAA | 168 |
| PsPP2A-fwd  PsPP2A-rev | CCACATTACCTGTATCGGATGACA  GAGCCCAGAACAGGAGCTAACA | 43 |
